# A pressure-resistant peripherally inserted central catheter is as useful as a central venous catheter for rapid fluid infusion: an in vitro study

**DOI:** 10.1186/s12871-022-01738-x

**Published:** 2022-07-04

**Authors:** Jun Maki, Makoto Sumie, Tomoko Ide, Masako Nagamatsu, Katsuyuki Matsushita, Kazuhiro Shirozu, Midoriko Higashi, Ken Yamaura

**Affiliations:** 1 Intensive Care Unit, Kyushu University Hospital, Fukuoka, Japan; 2 Department of Anesthesiology and Critical Care Medicine, Kyushu University Hospital, 3-1-1 Maidashi, Higashi-ku, Fukuoka, 812-8582 Japan

**Keywords:** Peripherally inserted central catheter (PICC), Central venous catheter (CVC), Fluid infusion, Rapid fluid infusion, Blood transfusion

## Abstract

**Background:**

Although peripherally inserted central catheters (PICCs) have been widely used, they have not been frequently used in anesthesia practice. The central venous pressure measured via PICCs are reportedly as accurate as that measured via central venous catheters (CVCs), but the findings concerning rapid infusion are unclear. This study examined whether or not pressure-resistant PICCs could be used for rapid fluid infusion.

**Methods:**

The in-line pressure was measured in similar-sized double-lumen catheters—4-Fr PICC (55, 45 and 35 cm) and 17-G CVC (20 and 13 cm)—at flow rates of saline decided using a roller pump system. We also examined the flow rate at an in-line pressure of 300 mmHg, which is the critical pressure at which hemolysis is considered to occur during blood transfusion.

**Results:**

The pressure-resistant PICCs obtained a high flow rate similar to that of CVCs, but the in-line pressures increased in proportion to the flow rate and catheter length. Flow rates at an intra-circuit pressure of 300 mmHg were not significantly different between the 45-cm PICC and 20-cm CVC.

**Conclusion:**

Pressure-resistant PICCs can be used for rapid fluid infusion.

**Supplementary Information:**

The online version contains supplementary material available at 10.1186/s12871-022-01738-x.

## Introduction

Central venous catheters (CVCs) are used in perioperative management to monitor central venous pressure (CVP), administer cardiovascular agents and drugs irritating to veins, perform rapid fluid infusion and transfuse blood. However, because CVC insertion is associated with severe mechanical complications, the indications of CVCs are limited [1]. In contrast, the insertion of a peripherally inserted central catheter (PICC) is safer and easier than that of a CVC according to a retrospective study [2]. Although PICCs are widely used for parenteral nutrition, chemotherapy, and long-term administration of antimicrobial agents, they have not been frequently used in perioperative management or acute care, as the accuracy of the CVP measured via a PICC and the reliability of PICCs as a route for rapid fluid infusion are unclear.

In hemodynamic monitoring, the CVP is measured via a CVC in 19% of non-cardiac surgeries lasting longer than 90 min and is useful for hepatobiliary surgeries [2, 3], kidney transplantation [4]. In both in vitro and clinical studies, the CVP measured via a PICC has been reported to be as accurate as that measured via a CVC [5, 6]. Therefore, the use of a PICC to monitor the CVP in anesthetic management and acute care is suggested to be possible.

As an intravenous route for rapid infusion and blood pressure, CVCs are also useful in clinical practice [7]. However, if PICCs are used as a rapid infusion route, in-line pressure may rise too much due to longer line route. In recent years, pressure-resistant PICCs suitable for injection of contrast media have become available. Therefore, in the present study, we examined whether or not pressure-resistant PICCs could be used for rapid fluid infusion as quickly as CVCs in vitro.

## Methods

The catheters examined were as follows: 55-cm 4-Fr (outer diameter 1.33 mm) dual-lumen PICC (Power PICC™; Bard Access Systems, Inc. Salt Lake City, UT, USA), and a 20- and 13-cm 17-G (outer diameter 1.35 mm) dual-lumen CVC (SMAC™ plus; Cardinal Health, Dublin, OH, USA) (Table 1). After measurement, the 55-cm PICC was cut to 45 and 35 cm, in sequence.

**Table 1:** Details of the PICC and CVC

	size	Number of lumens	OD (mm)	ID (G))		Length (cm)
				main lumen	second lumen	
PICC	4 Fr	2	1.33	19	21	55, 45, and 35
CVC	17 G	2	1.35	18	21	20 and 13

*PICC* Peripherally inserted central catheter, *CVC* Central venous catheter, *OD* Outer catheter diameter, *ID* Inner catheter diameter

**Fig. 1:**
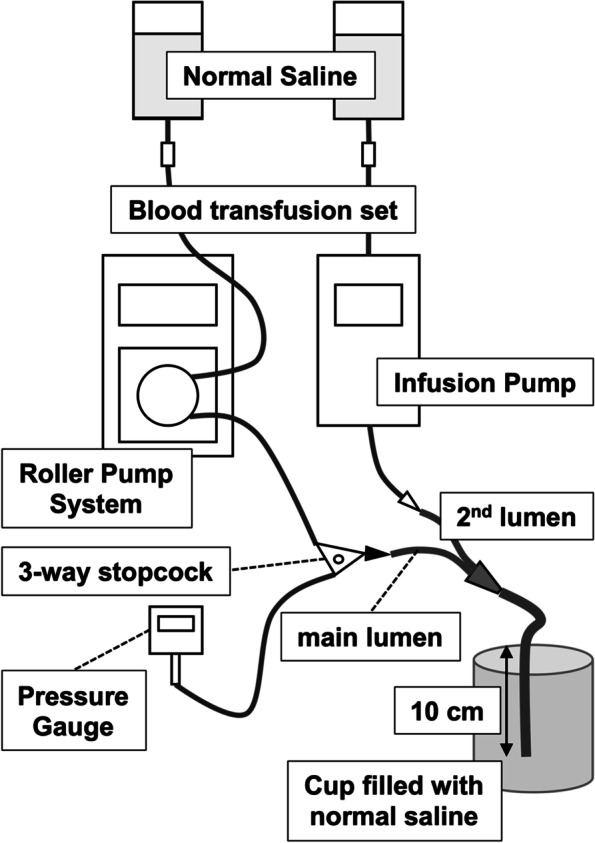
A schematic illustration of infusion circuit for measuring in-line pressure. The circuit to infuse normal saline consisted of a blood transfusion set, a roller pump system and a three-way stopcock connected to the main lumen of a catheter. A pressure gauge was connected to the stopcock. The second lumen of the catheter was perfused with normal saline using an infusion pump

Regarding the connection of the PICC and CVC to the infusion sets, the infusion circuit used to measure in-line pressure consisted of a blood transfusion set (TB-PU300L; TERUMO, Tokyo, Japan), the roller pump segment of a hemodialysis circuit (NS-1010–20; NIPRO, Osaka, Japan), and a three-way stopcock (L1-360FL; TOP, Tokyo, Japan). The blood transfusion set was connected to a 500-mL bag of normal saline solution, and the 3-way stopcock was connected to the main lumen of a dual-lumen catheter (a PICC or CVC). The circuit was attached to a roller pump system for hemodialysis (MP-301; NIPRO). A pressure gauge (PG-208-103GP-S; NIDEC COPAL ELECTRONICS, INC., Tokyo, Japan) was placed at the three-way stopcock to measure the in-line pressure. The second lumen of the catheter was connected to a bag of normal saline via another blood transfusion set and perfused with 20 mL/h normal saline using an infusion pump (TE-261; TERUMO), which simulated the use of a second lumen as a route for the administration of drugs such as catecholamines. The catheter tip was then placed 10 cm under the surface of normal saline solution in a cup open in the atmosphere (Fig. 1).

The flow rate of normal saline solution infused via the main lumen was regulated by the roller pump and increased and decreased stepwise by 10 mL/min between 0 and 150 mL/min. After the equilibration period, the maximum in-line pressure was recorded at each flow rate. The in-line pressure at each flow rate was measured four times in each catheter.

The data were expressed as the average ± standard error of the mean of values obtained from three catheters. The data were analyzed using a *t*-test or one-way analysis of variance with Tukey’s test. A *p*-value < 0.05 was considered statistically significant.

## Results

**Fig. 2:**
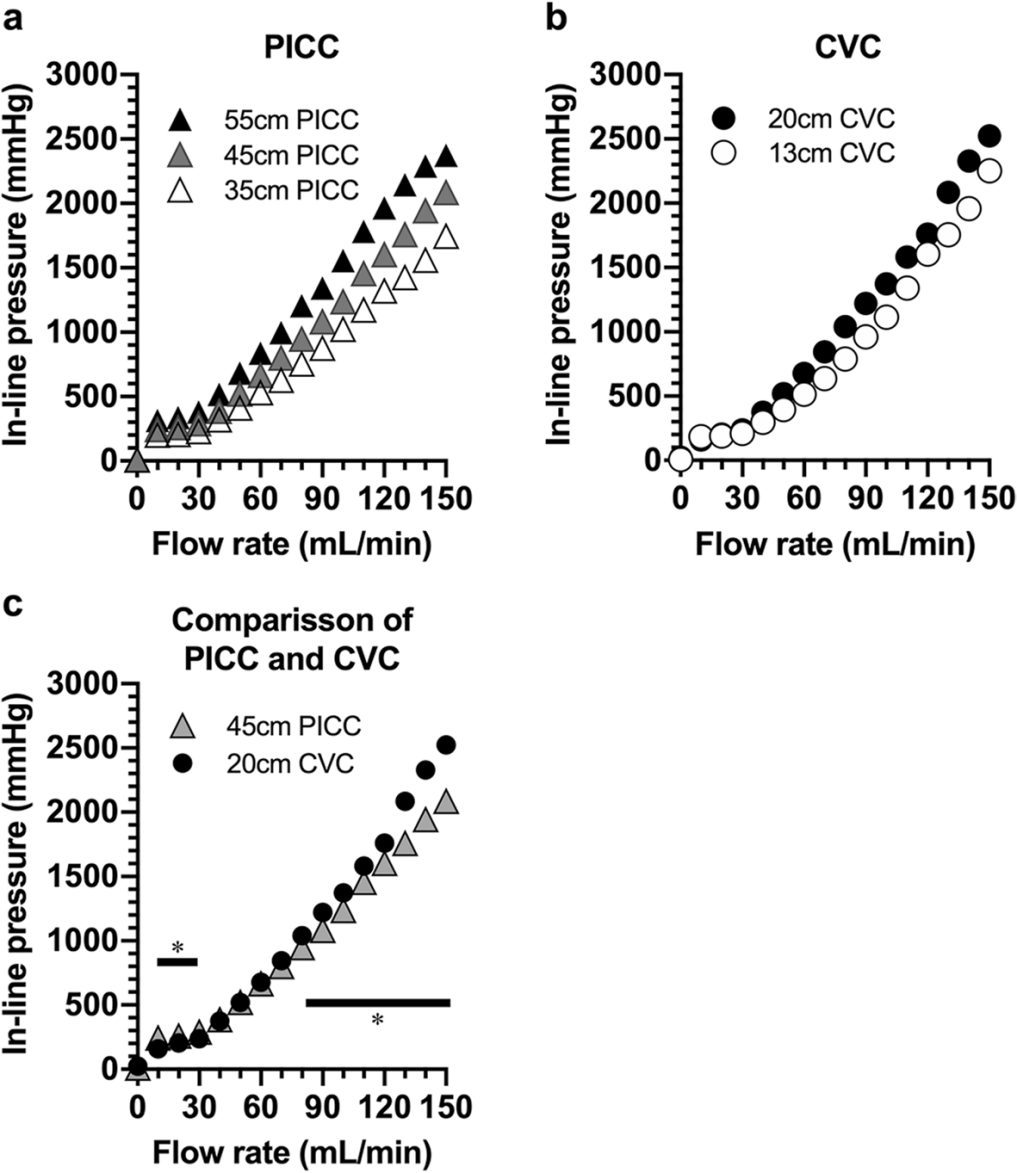
Relationships between the flow rates and in-line pressure. (**a**) Triangles and (**b**) circles show in-line pressures at indicated flow rates in 55-, 45- and 35-cm peripherally inserted central catheters (PICCs) and 20- and 13-cm central venous catheters (CVCs). (**c**) In-line pressures at the indicated flow rates in the 45-cm PICC (gray triangles) were compared with those in CVCs (filled circles). Data are shown as the average ± SEM, and significant differences are represented by * (*p* < *0.05*)

In-line pressures increased with the flow rate of normal saline at 0 to 150 mL/min in both the PICC and CVC (Fig. 2a, b). The maximum pressures in the 45-cm PICC and 20-cm CVC were 2084 ± 62 and 2523 ± 13 mmHg, respectively. In-line pressures increased in proportion to the length of catheter in both PICC and CVC (Fig. 2a, b). On comparing the 45-cm PICC and 20-cm CVC, the in-line pressures were higher in the 45-cm PICC at a low-flow range of 10 to 30 mL/min but lower in the 45-cm PICC at a high-flow range of 90 to 150 mL/min (Fig. 2c). Data comparing the 55-cm PICC and 20-cm CVC are shown in Additional file 2.

**Fig. 3:**
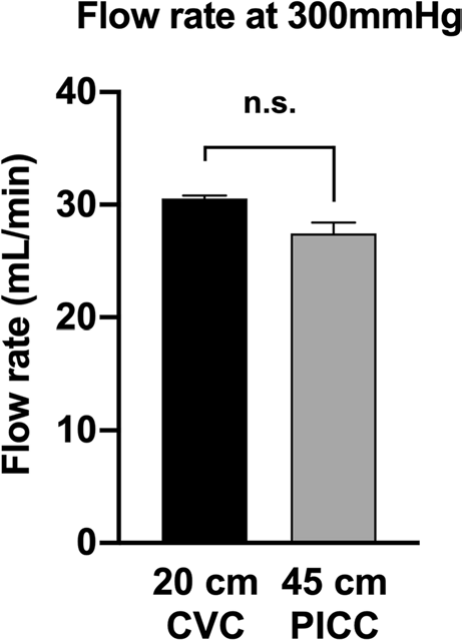
A comparison of the flow rates at an in-line pressure of 300 mmHg. The flow rates at an in-line pressure of 300 mmHg were compared between a 20-cm central venous catheter (CVC) and 45-cm peripherally inserted central catheter (PICC). The flow rates were not significantly different between these catheters (*p* < *0.05*). Data are shown as the average ± SEM. n.s., not significantly different

The flow rates when the in-line pressure was ≤ 300 mmHg decreased in proportion to the catheter length in both the PICC and CVC (data not shown). The flow rate when the in-line pressure was 300 mmHg was not significantly different between a 45-cm PICC and 20-cm CVC (Fig. 3) but was lower in the 55-cm PICC than in the 20-cm CVC (Additional file 3).

The in-line pressure was not affected by repeated measurement (Additional file 1).

## Discussion

In the present study, flow rates of 0–150 mL/min were obtained in the pressure-resistant PICC and CVC. However, the in-line pressures were positively correlated with the catheter length. The flow rate at in-line pressures < 300 mmHg, when hemolysis is expected to occur, did not markedly differ between the 45-cm pressure-resistant PICC and 20-cm CVC.

Regarding the relationship between the flow rate and in-line pressure, longer catheters have a higher in-line pressure than shorter ones. In our previous report concerning the relationship between the flow rate and in-line pressure using a peripheral venous catheter, there was a positive correlation between the catheter length and in-line pressure with an 18-G peripheral venous catheter, which has same diameter as the lumen of the PICC used in the present study. The in-line pressure was approximately 300 mmHg at a flow rate of 150 mL/min (9000 mL/h) [8]. The length of the peripheral venous catheter was 4.8 cm in the previous study, while the lengths of the CVC and PICC with the same diameter in the present study were 20 and 13 cm and 55, 45 and 35 cm. In each catheter, the in-line pressure increased to 1745–2523 mmHg as the catheter length was increased. However, in the present study, we confirmed that repeated infusions at an in-line pressure of ≥ 2000 mmHg did not affect the flow rate or in-line pressure using a pressure-resistant PICC or CVC. These results suggest that there is no issue with performing repeated high-pressure infusion.

Compared with rapid infusion from a peripheral venous catheter, the flow rate was limited when rapid intravenous infusion was performed with a PICC. However, a pressure-resistant PICC can withstand rapid intravenous infusion of 150 mL/min (9000 mL/h), which is clinically sufficient. The maximum pressure of a clinical pressurized rapid infusion device is limited to 300 mmHg, and it has been reported that in-line pressure is increased to about 600 mmHg during manual rapid infusion using a piston syringe [9]. In the present study, a pressure-resistant PICC was able to withstand a higher pressure than exerted with these methods, suggesting that a pressure-resistant PICC can be safely used for rapid infusion.

On comparing a 45-cm PICC with a 20-cm CVC, which are usually used clinically, the PICC, which was longer than the CVC, was expected to have a higher in-line pressure with the same flow rate provided the catheter diameter was the same. However, in actuality, the in-line pressure of the PICC was higher than that of the CVC at a low flow rate, whereas the in-line pressure of the PICC at a high flow rate was significantly lower than that of the CVC. This is considered to be due to the lumen partition wall of the double-lumen tube used in this study. In CVCs, the luminal septum does not move, whereas in pressure-resistant PICCs, the shape of the catheter lumen changes according to the in-line pressure, which expands the effective inner diameter of the catheter.

Regarding red blood cell products, hemolysis reportedly occurs under pressures exceeding 300 mmHg [10]. Thus, rapid transfusion using a pressurized rapid transfusion device was performed at a maximum internal pressure of 300 mmHg. In the present study, the rate of saline administration that resulted in an in-line pressure of 300 mmHg did not differ between the 45-cm PICC and 20-cm CVC and was comparable to 30 mL/min (1800 mL/h). Therefore, the pressure-resistant PICC can be used as a transfusion route as well as the CVC in case of rapid transfusion due to massive bleeding. If the flow rate is higher than this, transfusion should be performed through a larger venous route, such as a peripheral venous catheter or sheath.

Several limitations associated with the present study warrant mention. First, the data in the present study were obtained from an in vitro study. Thus, the in-line pressure may be influenced by the intravascular position and resistance of vasculature and may actually be much higher than the result shown here. Second, because the in-line pressure was not measured using red blood cells, the usefulness of a pressure-resistant PICC in blood transfusion has not been confirmed.

## Conclusion

Pressure-resistant PICCs can be used for rapid fluid infusion as fast as CVCs.

## Supplementary Information

**Additional file 1.** Changes in the in-line pressures after repeated measurements.

**Additional file 2.** In-line pressures at the indicated flow rates in the 55-cm PICC (gray triangles) were compared with those in CVCs (filled circles). Data are shown as the average ± SEM, and significant differences are represented by * (*p* < 0.05).

**Additional file 3.** A comparison of the flow rates at an in-line pressure of 300 mmHg between a 20-cm central venous catheter (CVC) and 55-cm peripherally inserted central catheter (PICC). The flow rates were significantly different between these catheters (*p* < 0.05). Data are shown as the average ± SEM. *, significantly different. 

## Data Availability

The datasets used and/or analyzed during the current study are available from the corresponding author on reasonable request. All data generated or analyzed during this study are included in this published article.

## Abbreviations

CVCs: Central venous catheters
CVP: Central venous pressure
PICCs: Peripherally inserted central catheter
