# Supplementary figures and images for: A pressure-resistant peripherally inserted central catheter is as useful as a central venous catheter for rapid fluid infusion: an in vitro study
Source: BMC Anesthesiol. 2022 Jul 4;22:205. doi: 10.1186/s12871-022-01738-x (PMC9252047; doi:10.1186/s12871-022-01738-x)

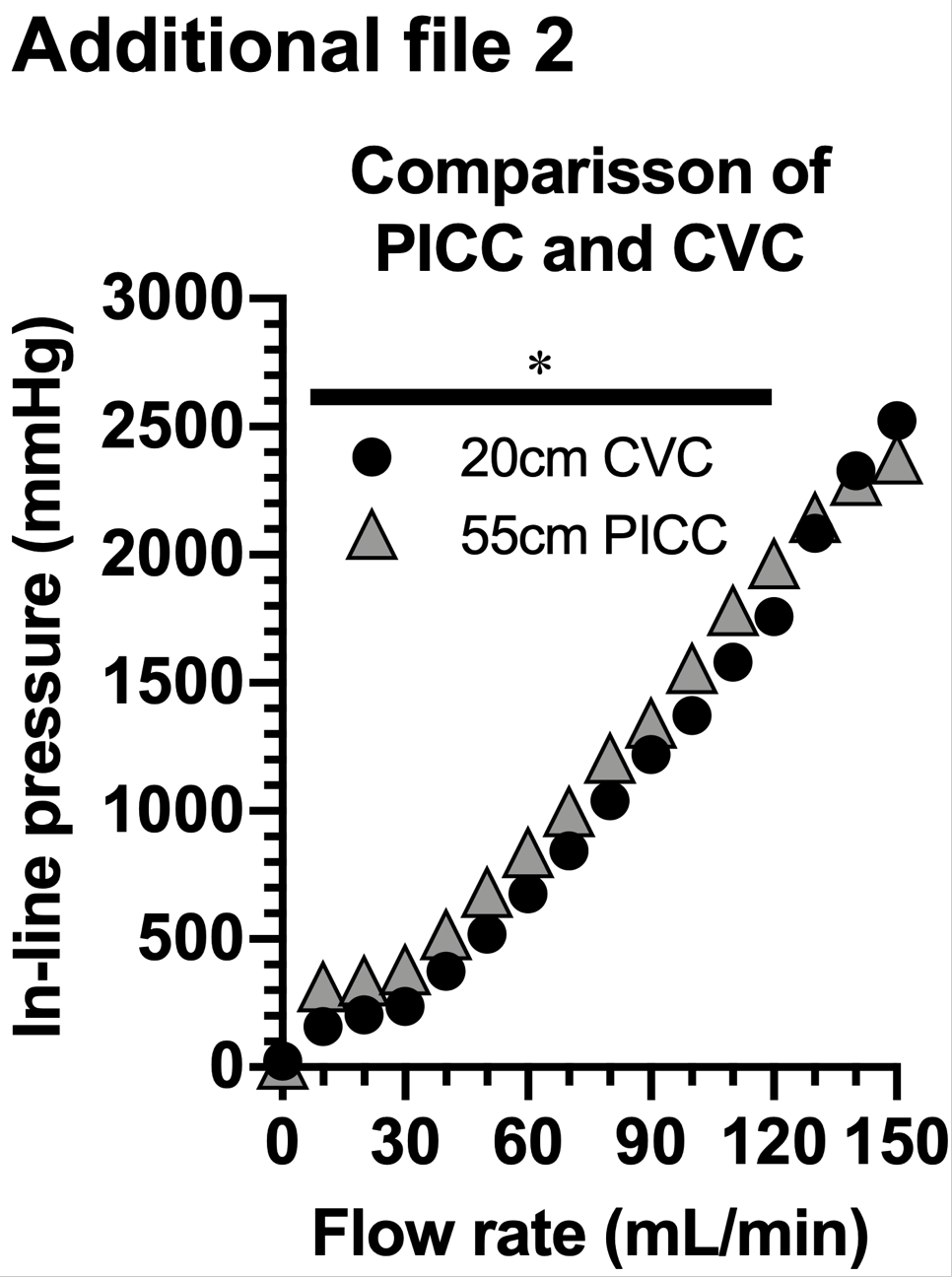

Supplement: Supplementary file 2 — Additional file 2. In-line pressures at the indicated flow rates in the 55-cm PICC (gray triangles) were compared with those in CVCs (filled circles). Data are shown as the average ± SEM, and significant differences are represented by * (p < 0.05). [file 12871_2022_1738_MOESM2_ESM.tiff]

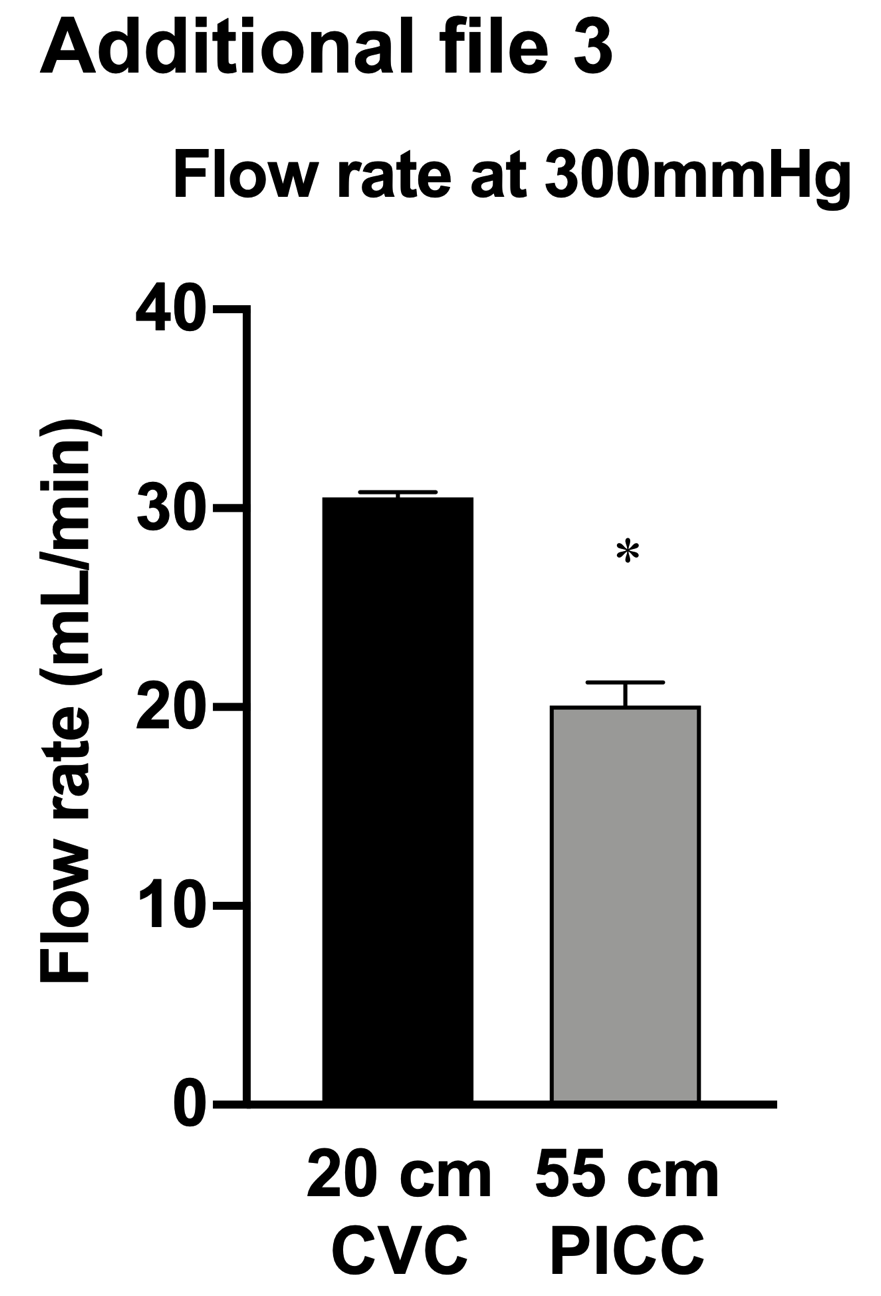

Supplement: Supplementary file 3 — Additional file 3. A comparison of the flow rates at an in-line pressure of 300 mmHg between a 20-cm central venous catheter (CVC) and 55-cm peripherally inserted central catheter (PICC). The flow rates were significantly different between these catheters (p < 0.05). Data are shown as the average ± SEM. *, significantly different. [file 12871_2022_1738_MOESM3_ESM.tiff]
